# Insulin resistance increases the occurrence of new cardiovascular events in patients with manifest arterial disease without known diabetes. The SMART study

**DOI:** 10.1186/1475-2840-10-100

**Published:** 2011-11-21

**Authors:** Sandra N Verhagen, Annemarie MJ Wassink, Yolanda van der Graaf, Petra M Gorter, Frank LJ Visseren

**Affiliations:** 1 Department of Vascular Medicine, University Medical Centre Utrecht (UMC Utrecht), Utrecht, The Netherlands; 2 Julius Centre for Health Sciences and Primary Care, UMC Utrecht, Utrecht, The Netherlands

**Keywords:** Insulin resistance, Manifest arterial disease, Metabolic syndrome

## Abstract

**Background:**

Insulin resistance is accompanied by a cluster of metabolic changes, often referred to as metabolic syndrome. Metabolic syndrome is associated with an increased cardiovascular risk in patients with manifest arterial disease. We investigated whether insulin resistance is associated with an increased risk for cardiovascular events in patients with manifest arterial disease without known diabetes and whether this can be explained by the components of the metabolic syndrome or by inflammation.

**Methods:**

Prospective cohort study in 2611 patients with manifest arterial disease without known diabetes. Homeostasis model of insulin resistance (HOMA-IR) was used to quantify insulin resistance. The relation of HOMA-IR with cardiovascular events (vascular death, myocardial infarction or stroke) and all cause mortality was assessed with Cox regression analysis. In additional models adjustments were performed for the single components constituting the metabolic syndrome and for inflammation.

**Results:**

HOMA-IR increases with the number of metabolic syndrome components (mean HOMA-IR ± SD in groups with 0, 1, 2, 3, 4 and 5 metabolic syndrome components: 1.4 ± 0.7; 1.8 ± 1.2; 2.4 ± 1.5; 3.1 ± 1.8; 4.0 ± 2.6; and 5.6 ± 3.6 respectively). High HOMA-IR was independently associated with an increased risk of cardiovascular events (tertile 2 vs. 1 HR 1.92; 95%CI 1.20-3.08) (tertile 3 vs.1 HR 1.78; 95%CI 1.10-2.89) and with all cause mortality (tertile 2 vs. 1 HR 1.80; 95%CI 1.04-3.10) (tertile 3 vs.1 HR 1.56; 95%CI 0.88-2.75). These relations were not influenced by the individual components of metabolic syndrome or by inflammation.

**Conclusions:**

In patients with manifest arterial disease without known diabetes, insulin resistance increases with the number of metabolic syndrome components, and elevated insulin resistance increases the risk of new cardiovascular events.

## Background

Both insulin resistance and metabolic syndrome are recognized as important factors in the development of cardiovascular disease [1,2]. Obesity-induced insulin resistance is considered to be the major driver of the clustering of interrelated metabolic disturbances (e.g. dyslipidemia, hyperglycemia, elevated blood pressure)[3], often referred to as metabolic syndrome [4], thereby leading to an increased cardiovascular risk. Although insulin resistance may be the unifying pathophysiological mechanism underlying the metabolic syndrome [5], there is uncertainty regarding the independent role of insulin resistance in the development of atherosclerotic vascular disease [1,5-12], also in patients with arterial diseases [13,14].

Metabolic syndrome is highly prevalent in patients with manifest arterial disease (46%)[15] and is associated with advanced vascular damage [16], thereby identifying those patients with an even higher cardiovascular risk. This high cardiovascular risk may be due to a combination of non-classical risk factors associated with insulin resistance, e.g. inflammation, hyperinsulinemia, oxidative stress, and hypercoagulability, together with the separate components of metabolic syndrome (low high-density lipoprotein (HDL)-cholesterol, elevated triglycerides, elevated glucose, elevated blood pressure, increased waist circumference)[17-19]. Nevertheless, the magnitude of the association between insulin resistance and the occurrence of new vascular events in these high-risk patients is not yet elucidated. In addition, it is not known whether insulin resistance *per se *has an influence on the elevated cardiovascular risk or that it is mediated by the components of metabolic syndrome.

Aim of the current study is (1) to investigate the relation between insulin resistance and metabolic disturbances, (2) to determine whether insulin resistance, derived by homeostasis model assessment of insulin resistance (HOMA-IR), is associated with increased occurrence of cardiovascular events and all cause mortality, and (3) to evaluate to what extent this relation can be explained by the individual components of the metabolic syndrome or by inflammation.

## Methods

### Study settings, participants and design

In this study, we used data from patients enrolled in the Second Manifestations of Arterial disease (SMART) study. The SMART study is an ongoing prospective single-centre cohort study in patients with manifest arterial disease or cardiovascular risk factors [20]. Started in September 1996, patients aged 18-80, were referred to the University Medical Centre (UMC) Utrecht with a recent diagnosis of manifest arterial disease or a cardiovascular risk factor. Not approached are patients with terminal malignant disease, those not independent in daily activities (Rankin scale > 3) or not sufficiently fluent in Dutch. Patients entered the SMART study if participation in the study was supported by the treating specialist and if the patients themselves consented to participate. The Medical Ethics Committee approved the study, and all participants gave their written informed consent. The rationale and design of the SMART study and a detailed description of the criteria used to define the different manifest arterial diseases were published previously [20].

For the current study, the data of the 3239 participants with clinical manifestations of arterial disease (coronary heart disease, cerebrovascular disease, peripheral arterial disease or abdominal aortic aneurysm) included between July 1, 2003 and March 1, 2010 were considered. A total of 548 patients with known Type 1 or Type 2 diabetes mellitus (T2DM) were not included in the analyses. Diabetes mellitus at baseline was defined as a referral diagnosis of diabetes, self-reported diabetes, or use of glucose lowering agents. If glucose was ≥ 7 mmol/l without a history of diabetes, patients were only included if they were not on glucose lowering therapy after 1 year follow up [21]. In addition, patients with missing data on glucose or insulin were excluded (n = 44).

### Data acquisition

All baseline measurements were performed on a single day at the UMC Utrecht. Medical history, use of current medication and current and past cigarette smoking behavior were derived from a questionnaire described elsewhere [20]. Height, weight, waist circumference and blood pressure were measured. Blood samples were collected after an overnight fast. Total cholesterol, triglycerides, HDL-cholesterol, creatinin, and high sensitive-C-reactive protein (hs-CRP) levels were measured. Hs-CRP measurements below the lower limit of detection of 0.2 mg/l (n = 45) were set at 0.2 mg/l. Plasma glucose was measured using commercial enzymatic dry chemistry kits (Johnson and Johnson). Plasma insulin was measured with an immunometric technique on an IMMULITE 1000 Analyzer (Diagnostic Products Corporation, Los Angeles, USA). Inter-assay coefficient of variation for insulin measurements was 9% at 7 mIU/l and < 5.5% at 20-120 mIU/l. Insulin measurements below the lower limit of detection of 2 mIU/l (n = 74) were set at 2 mIU/l.

HOMA-IR was used as quantitative estimate of the degree of insulin resistance at baseline. The value for insulin resistance can be assessed by the formula: HOMA-IR = (fasting serum glucose (mmol/l) × fasting serum insulin (mIU/l)/22.5)[22]. HOMA-IR correlates well with measurements obtained by means of the euglycemic clamp technique [23]. Therefore it provides a reliable approach to estimate insulin resistance and lends itself for use in large epidemiological studies [24]. In addition, quantitative insulin sensitivity check index (QUICKI) was measured to compare the results retrieved with HOMA. QUICKI was calculated with the formula QUICKI = 1/(log(insulin × glucose))[25].

Metabolic syndrome was defined according to the Adult Treatment Panel (ATP) III criteria [4]. Metabolic syndrome requires the presence of at least three of the following metabolic abnormalities: abdominal obesity (waist circumference > 102 cm in men and > 88 cm in women), high blood pressure (≥ 130 mmHg systolic or ≥ 85 mmHg diastolic), hypertriglyceridemia (serum triglycerides ≥ 1.70 mmol/l (150 mg/dl)), low HDL-cholesterol (serum HDL-cholesterol < 1.04 mmol/l (40 mg/dl) in men and < 1.29 mmol/l (50 mg/dl) in women), high fasting glucose (fasting serum glucose ≥ 5.6 mmol/l (100 mg/dl)). Patients on blood pressure-lowering medication were regarded as having high blood pressure.

### Follow up

All patients were asked to complete a questionnaire every 6 months. The questionnaire comprised of questions on hospital admissions and out-patient clinic visits in the preceding 6 months. If patients reported a possible event, hospital discharge letters and results of relevant laboratory results and radiology examinations were collected and evaluated by an Outcome Event Committee comprising of physicians from different departments. Outcome of interest for the present study was a composite endpoint of fatal or non-fatal cardiovascular events during follow-up. A cardiovascular event was defined as the occurrence of cardiovascular death, ischemic stroke or myocardial infarction (table 1). Forty-two out of 2611 patients (2%) were lost to follow-up. Duration of follow-up was defined as the period between the date of study inclusion and the date of the occurrence of a new cardiovascular event, date of loss to follow-up or the pre-selected closing date of March 1^st^, 2010.

**Table 1:** Definition of study outcome events

Stroke	Definite: relevant clinical features causing an increase in impairment of at least one grade on the modified Rankin scale, accompanied by an infarction on repeat brain imaging. Probable: clinical features that have caused an increase in impairment of at least one grade on the modified Rankin scale; without documentation by means of brain imaging.
Myocardial infarction	At least two of the following criteria: (I) chest pain for at least 20 min, not disappearing after administration of nitrates; (II) ST-elevation > 1 mm in two following leads or a left bundle branch block on the electrocardiogram; (III) Creatinine kinase (CK) elevation of at least two times the normal value of CK and a myocardial band-fraction > 5% of the total CK.
Vascular mortality	Death from ischemic or hemorrhagic stroke, congestive heart failure, myocardial infarction or rupture of abdominal aortic aneurysm. Sudden death: unexpected cardiac death occurring within 1 hour after onset of symptoms, or within 24 hours given convincing circumstantial evidence. Vascular death from other causes
Composite vascular outcome event	A composite of stroke, myocardial infarction and vascular mortality completed with a probable or definite retinal infarction or bleeding and probable or definite hemorrhagic stroke.
All-cause mortality	Death from any cause

### Data analysis

Baseline characteristics were reported across tertiles of HOMA-IR. Mean and standard deviation (SD) were reported if continuous variables were normally distributed and median and interquartile range if distributions were skewed. Differences between tertiles of HOMA-IR were tested with one-way ANOVA (continuous normal distributed variables) or Kruskal-Wallis test (continuous skewed variables).

A trend between the number of components of metabolic syndrome and HOMA-IR was investigated with ANCOVA (general linear model procedure), and adjusted for age and gender.

The hazard ratios (HR) with corresponding 95% confidence intervals (CI) for the occurrence of a new cardiovascular event and of total mortality associated with insulin resistance were estimated with Cox proportional hazards analysis. Three models were used to estimate the relation between HOMA-IR as independent variable and new cardiovascular events or total mortality as dependent variables. Model 1 was adjusted for age and gender. In model 2 additional adjustments were made for current smoking, use of lipid lowering medication and blood pressure lowering medication. In model 3 exploratory analyses are shown with additional adjustments for history of coronary artery disease, cerebrovascular disease, abdominal aortic aneurysm and peripheral arterial disease on top of model 2. Analyses were repeated with QUICKI as independent variable.

To investigate whether the relation between insulin resistance and cardiovascular disease can be explained by the single components of the metabolic syndrome or by inflammation, additional adjustments for waist circumference, HDL-cholesterol, triglycerides, fasting glucose, systolic blood pressure and hs-CRP were performed on top of model 2 (model 4). Significance was taken at the 5% level (two-sided).

To investigate whether the relation between HOMA-IR and new cardiovascular events is modified by other variables, interaction terms for age, gender, waist circumference and smoking were subsequently included in model 2. The p-values of these interaction terms were > 0.05 and it was concluded that no effect modification was present for these variables.

In the Cox regression analyses, single imputation methods were used to reduce missing covariate data for smoking (n = 3 (< 1%)), HDL-cholesterol (n = 4 (< 1%)), triglycerides (n = 2 (< 1%)), systolic blood pressure (n = 13 (< 1%)), waist circumference (n = 76 (3%)), hs-CRP (n = 11 (< 1%)), since incomplete case analysis leads to loss of statistical power and possibly bias.

## Results

### Study population

The mean age was 59.6 ± 10.4 years and 71% of patients were male. HOMA-IR levels were 2.9 ± 2.2 for males and 2.5 ± 1.8 for females. CIMT and incidence of albuminuria did not change substantially across tertiles of HOMA-IR. Further general characteristics of the study population according to tertiles of HOMA-IR are listed in table 2.

**Table 2:** Baseline characteristics of the study population (n = 2611) according to tertiles of HOMA-IR

HOMA-IR, tertiles	Tertile 1	Tertile 2	Tertile 3
HOMA-IR	1.1 ± 0.4	2.3 ± 0.4	4.9 ± 2.3
(Range)	0.4-1.7	1.7-3.0	3.0-23.3
	n = 872	n = 870	n = 869
Age (years)	59.4 ± 10.5	60.0 ± 10.4	59.4 ± 10.4
Male gender, n (%)	593 (68)	611 (70)	658 (76)
Body mass index (kg/m^2^)	24.7 ± 3.1	26.7 ± 3.4	28.9 ± 4.2
Abdominal adipose tissue (cm)	7.6 ± 2.0	8.7 ± 2.2	10.1 ± 2.5
Current smoking, n (%)	288 (33)	245 (28)	251 (29)
Creatinin clearance MDRD (ml/min/1.73 m2)	79.0 ± 16.8	75.4 ± 16.4	74.0 ± 18.3
			
*Medication*			
Lipid-lowering agents, n (%)	624 (72)	671 (77)	709 (82)
Anti-platelet agents, n (%)	689 (79)	723 (83)	724 (83)
Blood pressure-lowering agents, n (%)	588 (67)	654 (75)	751 (86)
			
*Location of manifest arterial disease*			
Cerebrovascular disease, n (%)	263 (30)	261 (30)	206 (24)
Coronary heart disease, n (%)	509 (58)	540 (62)	614 (71)
Peripheral arterial disease, n (%)	138 (16)	127 (15)	133 (16)
Abdominal aortic aneurysm, n (%)	56 (6)	64 (7)	44 (5)
			
*Atherosclerotic burden*			
CIMT(mm)	0.88 ± 0.24	0.89 ± 0.25	0.89 ± 0.22
Albumin/creatinin ratio > 3.0 mg/mmol, n (%)	120 (14)	120 (14)	135 (16)
			
*Metabolic syndrome components*			
Metabolic syndrome (ATPIII), n (%)	148 (17)	232 (37)	582 (67)
Waist circumference (cm)	87.6 ± 10.6	94.0 ± 10.5	100.5 ± 11.8
Blood pressure systolic (mmHg)	138 ± 20	141 ± 21	142 ± 21
Blood pressure diastolic (mmHg)	81 ± 11	83 ± 11	84 ± 11
Fasting glucose (mmol/l)	5.4 ± 0.5	5.7 ± 0.6	6.1 ± 0.8
Triglycerides (mmol/l)	1.0 (0.8-1.4)	1.2 (0.9-1.7)	1.1 (0.9-1.3)
HDL-cholesterol (mmol/l)	1.4 ± 0.4	1.3 ± 0.4	1.1 ± 0.3
			
LDL-cholesterol (mmol/l)	2.7 ± 0.9	2.7 ± 1.0	2.6 ± 0.9
hs-CRP (mg/l)	1.4 (0.6-3.2)	1.6 (0.8-3.7)	2.1 (1.0-4.3)
Fasting insulin (mIU/l)	4.8 ± 1.6	9.1 ± 1.5	17.9 ± 7.8
QUICKI	0.32 ± 0.05	0.26 ± 0.01	0.22 ± 0.02

### Insulin resistance and metabolic disturbances

Prevalence of metabolic syndrome was 42%. Metabolic syndrome was more prevalent in the highest HOMA-IR tertile compared with the lowest tertile (69% versus 16%) (table 2). Waist circumference, fasting glucose level, triglyceride level and systolic blood pressure were higher in patients within the highest HOMA-IR tertile, whereas HDL-cholesterol level was lower. Level of hs-CRP was incurred with increasing levels of insulin resistance. It is shown that HOMA-IR increases with an increment in the number of components of metabolic syndrome, adjusted for age and gender (p-value for trend < 0.001). (table 3)

**Table 3:** HOMA-IR in relation to metabolic syndrome (ATPIII) and the number of metabolic syndrome components in patients with manifest arterial disease

n = 2611		N	HOMA-IR	QUICKI	p-value
Metabolic syndrome	no	1478	2.1 ± 1.4	0.28 ± 0.05	< 0.001
	yes	1053	3.7 ± 2.5	0.24 ± 0.04	
					
Metabolic syndrome components	0	137	1.4 ± 0.7	0.31 ± 0.05	< 0.001
	1	547	1.8 ± 1.2	0.30 ± 0.06	
	2	794	2.4 ± 1.5	0.27 ± 0.05	
	3	573	3.1 ± 1.8	0.25 ± 0.04	
	4	366	4.0 ± 2.6	0.23 ± 0.03	
	5	114	5.6 ± 3.6	0.22 ± 0.03	

### Insulin resistance and occurrence of cardiovascular events

During a mean follow-up of 3.1 ± 1.9 years (total number of follow-up years 8094), 91 out of 2611 subjects died (of whom 47 due to a cardiovascular cause), 74 experienced a myocardial infarction and 34 an ischemic stroke. This corresponds with a cardiovascular event rate of 16 per 1000 patient years and a mortality rate of 11 per 1000 patient years. HOMA-IR was associated with an increased risk for the occurrence of a new vascular event, adjusted for age, gender, smoking and use of lipid lowering and blood pressure lowering medication (tertile 2 versus 1 HR 1.92 (95% confidence interval (CI) 1.20-3.08); (tertile 3 versus 1 HR1.78 (95%CI 1.10-2.89) (table 4)). Comparison of the lower tertiles of QUICKI with the highest tertile yielded exactly the same HRs as comparison of the higher HOMA-IR tertiles with the first tertile (table 5). Risk of death from any cause was increased in the second tertile of HOMA-IR versus the first (HR 1.80, (95%CI 1.05-3.10) and this risk was also increased, although non-statistically significant, in the third versus the first tertile (HR 1.56 (95%CI: 0.88-2.75).

**Table 4:** HOMA-IR and the risk for new cardiovascular events (vascular death, myocardial infarction or stroke) in patients with manifest arterial disease (n = 2611)

HOMA-IR		tertile 1		tertile 2		tertile 3	
	Model	# events	HR (95%CI)	# events	HR (95%CI)	# events	HR (95%CI)
All vascular events	1	26	1 (reference)	52	1.86 (1.16-2.98)	48	1.70 (1.05-2.74)
	2		1 (reference)		1.92 (1.20-3.08)		1.78 (1.10-2.89)
	3		1 (reference)		1.86 (1.16-3.00)		1.79 (1.10-2.90)
	4		1 (reference)		1.80 (1.10-2.96)		1.50 (0.85-2.68)
							
Myocardial infarction	1	17	1 (reference)	30	1.63 (0.90-2.96)	27	1.42 (0.77-2.61)
	2		1 (reference)		1.61 (0.89-2.93)		1.41 (0.76-2.61)
	3		1 (reference)		1.57 (0.86-2.85)		1.39 (0.75-2.59)
	4		1 (reference)		1.34 (0.72-2.51)		0.95 (0.46-1.98)
							
All cause mortality	1	20	1 (reference)	39	1.69 (0.99-2.91)	32	1.43 (0.82-2.51)
	2		1 (reference)		1.80 (1.05-3.10)		1.56 (0.88-2.75)
	3		1 (reference)		1.76 (1.02-3.03)		1.63 (0.92-2.88)
	4		1 (reference)		1.83 (1.04-3.24)		1.56 (0.78-3.09)
							
Vascular mortality	1	9	1 (reference)	23	2.26 (1.04-4.88)	15	1.50 (0.66-3.43)
	2		1 (reference)		2.42 (1.12-5.25)		1.72 (0.74-3.98)
	3		1 (reference)		2.24 (1.03-4.88)		1.74 (0.75-4.04)
	4		1 (reference)		2.56 (1.15-5.70)		1.85 (0.73-4.73)

Model 1: adjusted for age and gender

Model 2: model I additionally adjusted for current smoking, lipid lowering medication, blood pressure-lowering medication

Model 3: model II additionally adjusted for history of coronary artery disease, cerebrovascular disease, abdominal aortic aneurysm and peripheral arterial disease.

Model 4: model II additionally adjusted for waist circumference, systolic blood pressure, triglycerides, HDL-c, glucose and hs-CRP

**Table 5:** QUICKI and the risk for new cardiovascular events (vascular death, myocardial infarction or stroke) in patients with manifest arterial disease (n = 2611)

QUICKI		tertile 1		tertile 2		tertile 3	
	Model	# events	HR (95%CI)	# events	HR (95%CI)	# events	HR (95%CI)
All vascular events	1	48	1.70 (1.05-2.74)	52	1.86 (1.16-2.98)	26	1 (reference)
	2		1.78 (1.10-2.89)		1.92 (1.20-3.08)		1 (reference)
	3		1.79 (1.10-2.90)		1.86 (1.16-3.00)		1 (reference)
	4		1.50 (0.85-2.68)		1.80 (1.10-2.96)		1 (reference)
							
Myocardial infarction	1	27	1.42 (0.77-2.61)	30	1.63 (0.90-2.96)	17	1 (reference)
	2		1.41 (0.76-2.61)		1.61 (0.89-2.93)		1 (reference)
	3		1.39 (0.75-2.59)		1.57 (0.86-2.85)		1 (reference)
	4		0.95 (0.46-1.98)		1.34 (0.72-2.51)		1 (reference)
							
All cause mortality	1	32	1.43 (0.82-2.51)	39	1.69 (0.99-2.91)	20	1 (reference)
	2		1.56 (0.88-2.75)		1.80 (1.05-3.10)		1 (reference)
	3		1.63 (0.92-2.88)		1.76 (1.02-3.03)		1 (reference)
	4		1.56 (0.78-3.09)		1.83 (1.04-3.24)		1 (reference)
							
Vascular mortality	1	15	1.50 (0.66-3.43)	23	2.26 (1.04-4.88)	9	1 (reference)
	2		1.72 (0.74-3.98)		2.42 (1.12-5.25)		1 (reference)
	3		1.74 (0.75-4.04)		2.24 (1.03-4.88)		1 (reference)
	4		1.85 (0.73-4.73)		2.56 (1.15-5.70)		1 (reference)

Model 1: adjusted for age and gender

Model 2: model I additionally adjusted for current smoking, lipid lowering medication, blood pressure-lowering medication

Model 3: model II additionally adjusted for history of coronary artery disease, cerebrovascular disease, abdominal aortic aneurysm and peripheral arterial disease.

Model 4: model II additionally adjusted for waist circumference, systolic blood pressure, triglycerides, HDL-c, glucose and hs-CRP

### Influence of metabolic syndrome and inflammation on the relation between insulin resistance and cardiovascular risk

To investigate the independent relation between insulin resistance and cardiovascular events, additional analyses were performed adjusting for factors that are possibly in the causal pathway. Adjusting for the individual components of the metabolic syndrome did not substantially change the relation between HOMA-IR and the composite endpoint of all vascular events (tertile 2 versus 1 HR 1.76; 95%CI 1.08-2.89; tertile 3 versus 1 HR 1.48; 95%CI 0.83-2.63). Congruently adjustment for presence of the metabolic syndrome did not alter the point estimate for the relation between HOMA-IR and the composite endpoint (tertile 2 vs 1 HR 1.92 95%CI 1.20-3.08; tertile 3 vs1 (HR 1.78; 95%CI 1.10-2.90). Moreover, the relationship was not altered after adjusting for hs-CRP as a marker of inflammation (tertile 2 versus 1 HR 1.93; 95%CI 1.20-3.10; tertile 3 versus 1 HR 1.73; 95%CI 1.07-2.82). Hazard ratios for analyses with the single components of the metabolic syndrome and hs-CRP are depicted in table 4, model 4.

## Discussion

In patients with manifest arterial diseases without known diabetes, insulin resistance was elevated in patients with a higher number of metabolic syndrome components pointing towards a causal role of insulin resistance in the pathophysiology of metabolic syndrome. Furthermore, in this population, insulin resistance is associated with an increased risk to develop a second vascular event, independent of risk factors clustering in the metabolic syndrome including inflammation. Insulin resistance also tended to be associated with an increased risk of death form any cause, although the relationship between insulin resistance and all-cause mortality was not statistically significant in the highest tertile of HOMA-IR

These results imply that insulin resistance is a major driver underlying the clustering of metabolic abnormalities and that insulin resistance is also directly and independently related to an increased risk for cardiovascular events in patients with manifest arterial disease. In general, patients with insulin resistance are at increased cardiovascular risk [1,5-12]. Two other studies have shown that insulin resistance is associated with an increased risk for new vascular events in patients with manifest arterial disease [13,14]. Furthermore HOMA-IR has been found to independently predict the occurrence of a secondary event in patients with acute coronary syndrome [26]. In these studies, HOMA-IR was lower and the range was less broad as compared to the present study. The reason for the relatively high HOMA-IR in our cohort is not fully clear and not attributable to high BMI, since BMI in our study population was comparable or even lower than in these previous studies.

Obesity is associated with the metabolic abnormalities and an increased blood pressure clustered in the metabolic syndrome [3]. With an increase in adipocyte size, capillary density in adipose tissue decreases by an increase in collagen IV in the capillary walls, preventing angiogenesis in vitro [27]. Oxidative stress caused by a decreased capillary density may be the underlying mechanism of adipose tissue dysfunction characterized by secretion of pro-inflammatory cytokines and metabolic changes [28]. As a result of adipose tissue dysfunction efflux of free fatty acids increases, eventually giving rise to systemic dyslipidemia [29] One of the potential mechanisms for obesity-associated hypertension linking hypertension and insulin resistance is a decrease in cardiac natriuretic peptides as observed in insulin resistance [30].

Results of the present study are clinically relevant, since improving insulin sensitivity may improve cardiovascular outcome, as was already suggested in patients with type 2 diabetes [31,32]. In a randomized trial investigating the optimal treatment in diabetic patients with stable ischemic heart disease, there was a trend towards a lower cardiovascular risk for insulin sensitization compared to insulin provision in the group of patients randomized to undergo revascularization as opposed to medical therapy alone [32]. Improving insulin sensitivity with the peroxisome proliferator-activated receptor (PPAR-)γ agonist pioglitazone was demonstrated to reduce the occurrence of macrovascular events in patients with type 2 diabetes [31].

The increased occurrence of cardiovascular events in patients with insulin resistance is most likely due to atherosclerotic disease. Arterial stiffness is considered to be an early manifestation of atherosclerosis. In non-diabetic subjects, fasting glucose was associated with arterial stiffness as measured with pulse wave velocity [33]. In patients without diabetes, pulse wave velocity was an independent predictor for intima media thickness, but not in patients with diabetes [34]. The mechanisms by which insulin resistance leads to atherosclerotic cardiovascular disease are not fully understood. Indirect effects through accelerating chronic inflammation, oxidative stress, and metabolic abnormalities (e.g. hyperglycemia, dyslipidemia, and elevated blood pressure) have been implicated to contribute to endothelial dysfunction, thereby enhancing atherosclerotic cardiovascular disease [17,35]. In patients with manifest arterial disease the single components of the metabolic syndrome did not increase the risk of new vascular events in contrast to these components combined in the metabolic syndrome [36]. The metabolic syndrome is under debate because of its limited additive value in prediction of cardiovascular disease on top of classical risk factors [37]. However, from an etiologic point of view, the concept of the metabolic syndrome helps us to understand the pathophysiology of insulin resistance and its related metabolic changes leading to an increased cardiovascular risk. In the present study we have evaluated the etiologic relation between insulin resistance and cardiovascular risk. Metabolic syndrome is highly related to insulin resistance.

In our analyses, adjusting for the individual components of the metabolic syndrome did not alter the relation between insulin resistance and the risk of new vascular events. This implies that, besides the components constituting the metabolic syndrome, there are other factors that drive the relationship between insulin resistance and the risk of new cardiovascular endpoints. In adipose tissue dysfunction adipocytes enlarge giving rise to insulin resistance and an increase in adipocytokine production leading to a pro-inflammatory state. However, our analyses do not point to inflammation as a key factor in the relationship between insulin resistance and vascular disease as adjustment for hs-CRP did not substantially influence the point estimate for the risk of cardiovascular events across tertiles of HOMA-IR. Besides pro-inflammatory effects, adipocytokines are known to induce pro-haemostatic, pro-coagulant effects [38], although platelet aggregation is reduced in hyperinsulinemic states [39]. Furthermore endothelial function is impaired in insulin resistance through imbalance of nitric oxide production and the release of vasoconstrictor endothelin-1[40]. Various adipocytokines may also directly affect endothelial function and accelerate processes involved in atherogenesis. Another explanation may be the change in lipoprotein particle composition. Insulin resistance is associated with the pro-atherogenic small dense LDL-cholesterol concentration [41].

We acknowledge some limitations of our study. Compared to other studies with comparable patient groups, there was a relatively low mortality rate, limiting the statistical power of the study. HOMA-IR was used to estimate the level of insulin resistance. Although the hyperinsulinemic euglycemic clamp technique is the standard for measuring insulin resistance [42], this technique is unsuitable for epidemiologic studies. HOMA-IR seems to be a reliable tool in the assessment of insulin resistance due to its strong relation to clamp-measured insulin resistance in both patients with and without diabetes [23,24]. Moreover, repeating analyses with the QUICKI index as a measure of insulin sensitivity confirmed the results retrieved with HOMA-IR. It can be agued that it is not correct to include glucose in models together with HOMA-IR. However since hyperglycemia is one of the effects of insulin resistance and part of the metabolic syndrome just as dyslipidemia and hypertension, we decided to adjust for all components of the metabolic syndrome including glucose. Moreover, there is no single clinical definition for the clustering of metabolic abnormalities. We used the ATPIII-definition of metabolic syndrome [4] because it is most commonly used in studies, best related with the development of vascular diseases and easy to use in clinical practice. However, we realize that there are more definitions for the metabolic syndrome [3].

In conclusion, in patients with manifest arterial disease without diabetes, insulin resistance clusters with the number of components of the metabolic syndrome, and elevated insulin resistance is independently associated with an increased risk for cardiovascular events. This relation can not be explained by the single components constituting the metabolic syndrome or by inflammation.

## Competing interests

The authors declare that they have no competing interests.

## Authors' contributions

All authors have read and approved the final manuscript. SV carried out the analyses, had an important part in the conception of the present study, interpretation of the data and in writing the manuscript. YG participated in the design of the SMART-study and critically reviewed the analyses and the design of the present study. AW participated in performing the analyses and critically reviewed the manuscript. PG participated in the conception of the present study and made a substantial contribution in drafting the manuscript. FV made substantial contributions in interpretation of the data, conception of the present study and in writing the manuscript.
